# Colorectal Cancer Risk Following Herpes Zoster Reactivation in COVID-19 Survivors: Global Multicenter Study Using TriNetX

**DOI:** 10.3390/cancers17142306

**Published:** 2025-07-11

**Authors:** Tzung-Ju Lu, Chien-Lin Lu, Joshua Wang, Kuo-Wang Tsai, I-Hung Chen, Kuo-Cheng Lu

**Affiliations:** 1 Division of Colon and Rectal Surgery, Department of Surgery, Taipei Tzu Chi Hospital, Buddhist Tzu Chi Medical Foundation, New Taipei City 23142, Taiwan; 2 School of Medicine, Tzu Chi University, Hualien 970374, Taiwan; 3 Division of Nephrology, Department of Internal Medicine, Fu Jen Catholic University Hospital, School of Medicine, College of Medicine, Fu Jen Catholic University, New Taipei City 242062, Taiwan; 4 Department of Research, Taipei Tzu Chi Hospital, Buddhist Tzu Chi Medical Foundation, New Taipei City 23142, Taiwan; 5 School of Biomedical Sciences, Queensland University of Technology, Brisbane, QLD 4001, Australia; 6 Department of Nursing, Cardinal Tien Junior College of Healthcare and Management, New Taipei City 23143, Taiwan; 7 Department of Internal Medicine, Tri-Service General Hospital, National Defense Medical Center, Taipei City 11490, Taiwan; 8 Institute of Medical Science and Technology, National Sun Yat-sen University, Kaohsiung City 80424, Taiwan; 9 Department of Internal Medicine, Kaohsiung Armed Forces General Hospital, Kaohsiung City 80284, Taiwan; 10 Division of Nephrology, Department of Medicine, Taipei Tzu Chi Hospital, Buddhist Tzu Chi Medical Foundation, New Taipei City 23142, Taiwan; 11 Division of Nephrology, Department of Medicine, Fu Jen Catholic University Hospital, School of Medicine, Fu Jen Catholic University, No. 289, Jianguo Rd., Xindian Dist., New Taipei City 23142, Taiwan

**Keywords:** colorectal cancer, COVID-19, herpes zoster

## Abstract

Background: COVID-19 has been linked to prolonged immune dysfunction and long-term health complications. Herpes zoster (HZ), a marker of impaired cell-mediated immunity, may signal increased vulnerability to infections, cardiovascular disease, and potentially cancer. However, its association with colorectal cancer (CRC) after COVID-19 has not been fully explored. Objective: To investigate the long-term risks of cardiovascular events, acute respiratory failure, sepsis, and CRC in COVID-19 survivors who developed HZ compared to those who did not. Methods: We conducted a retrospective cohort study using the TriNetX Global Collaborative Network. Adults diagnosed with COVID-19 between January 2020 and January 2022 were included. Among the full cohort (aged ≥18 years), 27,664 patients with post-COVID HZ were identified. Due to platform limitations, propensity score matching (PSM) was applied to a restricted subgroup of patients aged 55–60 years, yielding a 1:1 matched cohort for controlled comparisons. Outcomes were assessed over a three-year follow-up. Results: In the matched age-restricted cohort, patients with post-COVID HZ had significantly higher risks of cardiovascular events, acute respiratory failure, sepsis, and CRC compared to matched controls. Subgroup analyses identified age ≥ 50, chronic kidney disease, diabetes, and hypertension as strong independent risk factors across outcomes. Despite the low absolute CRC incidence, cancer-free survival significantly favored the non-HZ group. Conclusion: Herpes zoster reactivation after COVID-19 is associated with increased risk of colorectal cancer. Enhanced surveillance and early CRC screening may benefit this high-risk population.

## Simple Summary

COVID-19 can weaken the body’s immune system, allowing dormant viruses like the one that causes shingles to become active again. This study investigated whether people who developed shingles after recovering from COVID-19 were more likely to face serious long-term health issues, including heart disease, infections, and colorectal cancer. Using a large international health database, we analyzed tens of thousands of patients and found that those with shingles after COVID-19 had significantly higher health risks. These findings suggest that shingles could be a warning sign of lingering immune problems. Doctors should consider closer monitoring and earlier cancer screening for this group to improve long-term health outcomes.

## 1. Introduction

Coronavirus Disease 2019 (COVID-19), caused by the Severe Acute Respiratory Syndrome Coronavirus 2 (SARS-CoV-2), is associated with a wide range of long-term complications, collectively known as post-acute sequelae of SARS-CoV-2 infection (PASC) [1]. Recent research has begun to examine the broader consequences of COVID-19, particularly its effects on the immune system and the potential heightened risk of cancer development following infection. Although direct causality is still being investigated, accumulating evidence indicates that SARS-CoV-2 may play a role in oncogenic processes through mechanisms such as immune dysregulation, chronic inflammation, and cellular stress [2].

Cancer patients have been identified as more susceptible to SARS-CoV-2 infection, with several immune pathways being investigated and documented [3]. Individuals recovering from COVID-19 often exhibit T-cell exhaustion, a reduction in natural killer (NK) cells, and persistent systemic inflammation. These factors may compromise immune surveillance, the body’s natural defense against the transformation of normal cells into malignant ones [4]. This raises the potential that SARS-CoV-2 may selectively infect or alter the microenvironment of tissues already vulnerable to oncogenic transformation [5]. Some studies have also suggested that viral infections may influence tumor suppressor pathways, such as p53 and retinoblastoma (Rb), potentially accelerating carcinogenesis [6].

Co-infection and superinfection with COVID-19 are increasingly recognized and clinically significant conditions that have garnered considerable attention. Evidence suggests that viral co-infections can significantly worsen the outcomes of COVID-19 patients. Recent studies indicate that viral superinfections may play a major role in the clinical deterioration of COVID-19 cases [7]. The immune dysregulation induced by SARS-CoV-2, combined with treatments such as mechanical ventilation and corticosteroid use, creates an environment conducive to secondary viral replication [7,8]. A recent study highlighted the growing occurrence of secondary viral reactivations, particularly herpes zoster (HZ), among COVID-19 survivors, driven by immune dysregulation following the viral infection [9,10].

Although HZ is increasingly recognized as a clinical marker of impaired cellular immunity, its association with malignancies—particularly colorectal cancer (CRC)—remains understudied. A population-based case-control study from the UK reported that patients with prior HZ had significantly increased risks of multiple cancers, including colorectal cancer (HR > 2.0), suggesting that HZ may reflect deeper immune compromise predisposing to oncogenesis [11]. In contrast, a more recent cohort study found no increased risk of overall cancer following HZ, except in patients who had postherpetic nervous system involvement, where a modest elevation in cancer risk, including CRC, was noted. These findings suggest that the oncologic significance of HZ may depend on the severity or extent of reactivation-related immune dysfunction [12].

Given the growing global burden of COVID-19 survivors and the increasing recognition of herpes zoster (HZ) as both a potential post-viral complication and a clinical marker of immunologic vulnerability [13,14], it is of particular interest to explore whether viral superinfection may play a role in the incidence of colorectal cancer. Understanding this association is crucial, as it has significant implications for colorectal cancer screening strategies. CRC is among the most common malignancies worldwide, and its prognosis greatly depends on early detection. During the COVID-19 pandemic, substantial declines in CRC screening and diagnosis were reported, making CRC a particularly relevant model for studying the combined effects of immune dysfunction and healthcare disruption.

This study aims to evaluate whether COVID-19 survivors who develop herpes zoster (HZ) during the post-infection period are at increased risk for mortality, cardiovascular events, renal complications, and colorectal cancer. HZ is investigated as a clinical marker of post-viral immune vulnerability, rather than a direct mediator, to assess its potential role in identifying high-risk individuals. Using a large, real-world multicenter dataset and rigorous propensity score matching, the study seeks to identify this high-risk subgroup. The findings are expected to improve post-COVID risk profiling and inform targeted prevention and management strategies, including the early screening for colorectal malignancies.

## 2. Materials and Methods

### 2.1. Study Design and Data Source

This retrospective cohort study utilized data from TriNetX, a global federated health research network providing access to deidentified electronic health records (EHRs) from a wide range of healthcare organizations. Specifically, the study used data from the Global Collaborative Network within TriNetX, which includes 144 healthcare organizations worldwide. TriNetX captures comprehensive structured patient data, such as demographics, diagnoses, procedures, medications, and laboratory values.

The analysis, conducted on 14 May 2025, was performed using the Compare Outcomes module within the TriNetX platform, ensuring compliance with the Health Insurance Portability and Accountability Act (HIPAA) and the General Data Protection Regulation (GDPR). The study follows the Strengthening the Reporting of Observational Studies in Epidemiology (STROBE) guidelines for observational research. Ethical approval was obtained from the Institutional Review Board of Taipei Tzu Chi Hospital (approval number: 14-IRB043).

### 2.2. Study Population

This study included patients identified from the TriNetX Global Collaborative Network who had a confirmed diagnosis of COVID-19 in adulthood (aged ≥18 years at diagnosis) between 1 January 2020 and 1 January 2022. COVID-19 was defined by either a positive SARS-CoV-2 RNA laboratory result (TNX:9088) or a clinical diagnosis coded as ICD-10-CM U07.1. Patients were subsequently categorized based on the occurrence of HZ within a defined post-COVID time window. This study initially identified 10,089,902 adults (aged ≥18 years) diagnosed with COVID-19.

**Figure 1:**
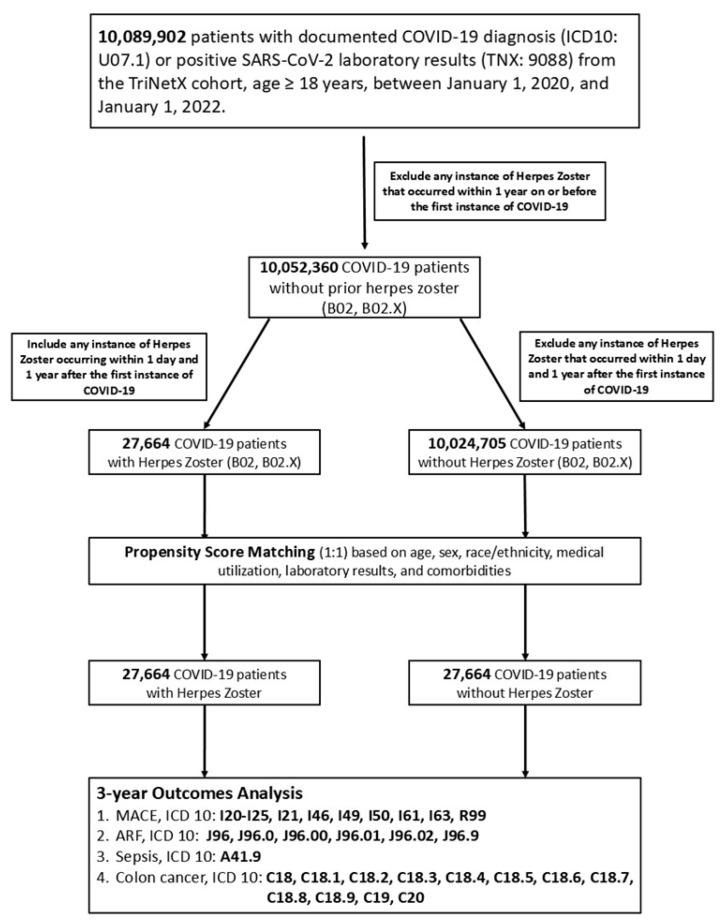
Flowchart describing patient selection, inclusion, and exclusion criteria. From a total of 10,052,360 eligible adult COVID-19 patients, two groups were defined based on the presence or absence of herpes zoster (HZ) during the 1-year post-COVID follow-up: (1) patients who developed HZ between 1 and 365 days after COVID-19 diagnosis (*n* = 27,664); (2) patients with no documented HZ during this time window (*n* = 10,024,705). Patients with HZ prior to or on the date of COVID-19 diagnosis were excluded.

Patients with any instance of HZ occurring within one year prior to or on the day of COVID-19 diagnosis were excluded, resulting in 10,052,360 eligible individuals. Patients who had no documented HZ diagnosis during this interval were classified as having COVID-19 without HZ. Diagnoses of HZ were determined using ICD-10-CM codes B02, B02.1, B02.2, B02.3, B02.7, B02.8, and B02.9. These were then stratified into two cohorts: Cohort 1 included patients who developed HZ between 1 day and 1 year after COVID-19 diagnosis, while Cohort 2 comprised those without HZ during the same time window. In the full adult cohort (aged ≥18 years), we identified 27,664 patients with post-COVID herpes zoster and 10,024,705 patients without herpes zoster, as shown in Figure 1.

The study evaluated four key outcomes over a 3-year follow-up: major adverse cardiovascular events (MACE; ICD-10: I20–I25, I21, I46, I49, I50, I61, I63, R99), acute respiratory failure (ARF) (ICD-10: J96, J96.0, J96.00, J96.01, J96.02, J96.9), sepsis (ICD-10: A41.9), colorectal cancer (ICD-10: C18, C19, C20). This design enables robust comparisons of long-term risks associated with HZ among COVID-19 survivors, providing clinically meaningful insights for risk stratification, surveillance, and preventive care in vulnerable populations.

### 2.3. Index Date and Follow-Up Period

The index date was defined as the first instance of a COVID-19 diagnosis or a positive laboratory result. Each patient’s observation period started the day after the index date and lasted for up to 3 years (1095 days), or until death or loss to follow-up, whichever occurred first. Outcomes occurring before the index date or within the first day were excluded from the risk analysis.

### 2.4. Outcome Measures

The primary outcomes assessed in this study were acute respiratory failure, MACE, sepsis, and the cumulative incidence-free probability of colorectal cancer (by KM analysis). Acute respiratory failure was identified either through a recorded deceased status in the demographic records or by a diagnosis code (ICD-10: J96.X). MACE was defined as a composite endpoint comprising acute myocardial infarction (I21), cardiac arrest (I46), other cardiac arrhythmias (I49), heart failure (I50), nontraumatic intracerebral hemorrhage (I61), cerebral infarction (I63), and ischemic heart diseases (I20–I25), or death (R99). Renal function decline was defined as eGFR < 60 mL/min/1.73 m^2^, based on the most recent value during the observation window, calculated using the CKD-EPI 2021 formula (UMLS: LNC: 98979-8). Colorectal cancer events were defined as a composite endpoint based on ICD-10-CM codes C18, C19, and C20. To ensure that only incident cases were included, patients with any documented CRC diagnosis prior to or on the index date were excluded from all outcome analyses. This approach ensured temporal clarity between exposure and outcome and avoided inclusion of prevalent, recurrent, or metastatic cases.

### 2.5. Propensity Score Matching (PSM) in an Age-Restricted Group

Due to computational constraints associated with the large-scale dataset within the TriNetX platform, it was not feasible to extract and perform detailed analyses on the baseline characteristics for the entire HZ cohort (*n* = 27,664). Therefore, we selected a representative age-restricted subgroup of patients aged 55 to 60 years, drawn from within the 27,664-patient post-COVID HZ cohort, comprising 3155 individuals with post-COVID HZ and 963,799 without HZ. This subgroup was drawn from the broader adult cohort and chosen to ensure feasibility while maintaining epidemiological relevance for age-related outcomes such as colorectal cancer.

A 1:1 propensity score matching (PSM) was performed within the age-restricted subgroup (55–60 years) to minimize baseline imbalances and reduce confounding. Matching was conducted using a greedy nearest-neighbor algorithm based on 8 key characteristics: age at index, female, male, White, Black or African American, Asian, diabetes mellitus, and hypertensive diseases. These covariates were chosen for their clinical relevance, potential to confound observed relationships, and potential to influence both herpes zoster susceptibility and long-term outcomes. Covariate balance was assessed using standardized mean differences, with values <0.1 considered indicative of adequate matching quality. Full post-matching characteristics are now presented in Table 1. This table includes all demographic, diagnostic, medication, and laboratory covariates.

Before matching, significant differences were observed between the COVID+HZ group (*n* = 3155) and the much larger COVID-HZ group (*n* = 963,799). The HZ group had a significantly higher proportion of females (66.5%) and White individuals (71.0%), compared to the non-HZ group (*n* = 963,799), which had 54.4% females and 57.9% White individuals. These differences were statistically significant (*p* < 0.001), indicating demographic imbalance. After 1:1 PSM, all demographic differences were eliminated, demonstrating successful matching. Patients’ comorbidities are also checked. Before matching, the COVID+HZ group exhibited higher rates of diabetes mellitus (14.3% vs. 7.5%) and hypertension (25.4% vs. 15.0%), both *p* < 0.001, suggesting a higher baseline burden of cardiometabolic disease. After matching, the groups were balanced, with no significant differences.

For patient medication use, the HZ group had significantly higher pre-matching rates of cardiovascular and metabolic medication prescriptions, including beta blockers (9.3% vs. 5.7%, *p* < 0.001), diuretics (11.0% vs. 6.4%, *p* < 0.001), and antilipemic agents (13.2% vs. 8.3%, *p* < 0.001), indicating a higher baseline burden of chronic disease. After propensity score matching, most medication imbalances were reduced. However, small residual differences persisted for beta blockers (9.2% vs. 9.2%, *p* = 0.862) and antilipemic agents (13.2% vs. 13.1%, *p* = 0.941), which may reflect either true associations or residual confounding. Laboratory data were also reviewed. Before matching, most laboratory values were broadly similar between groups, although small but statistically significant differences were observed in hemoglobin (13.3 ± 2.0 vs. 13.5 ± 2.0 g/dL, *p* < 0.001), serum ferritin (368.3 ± 767.2 vs. 290.1 ± 828.5 ng/mL, *p* = 0.045), and creatinine (1.1 ± 1.3 vs. 1.0 ± 1.4 mg/dL, *p* = 0.022), suggesting minor baseline variations in anemia, inflammation, or renal status. After matching, these differences were attenuated and no longer statistically significant. Other laboratory values, including iron and creatinine levels, remained largely comparable, supporting adequate biological balance between groups.

To ensure the robustness and generalizability of our findings, we performed parallel three-year outcome analyses by comparing hazard ratios for major adverse cardiovascular events, acute respiratory failure, sepsis, and colorectal cancer incidence between the age-restricted matched cohort (aged 55 to 60 years) and the full cohort (aged ≥18 years). As shown in Supplementary Table S1, the similarity in outcome risk profiles supports the assumption that the age-restricted matched cohort is representative of the broader adult population. This, in turn, enhances the interpretability and external validity of our findings derived from the matched analysis.

### 2.6. Statistical Analyses

Following propensity score matching, absolute risks, risk differences, risk ratios, and odds ratios (ORs) for each outcome were calculated using the TriNetX Risk Analysis module. Patients with prior occurrences of the outcome before the index event were excluded from the risk calculations. Kaplan–Meier survival analyses were then performed to estimate cumulative incidence and survival probabilities. Survival curves between cohorts were compared using the log-rank test, and hazard ratios (HRs) with 95% confidence intervals (CIs) were calculated using Cox proportional hazards models to assess time-to-event differences. For the primary outcome comparisons, statistical significance was assessed at a two-tailed *p*-value threshold of <0.05.

Multiple subgroup analyses were conducted to explore potential effect modification across age, comorbidities, laboratory markers, and other clinical covariates. However, no formal correction for multiple comparisons (e.g., Bonferroni or false discovery rate adjustment) was applied, given the exploratory nature of these analyses. Accordingly, the results from subgroup analyses should be interpreted as hypothesis-generating rather than confirmatory.

All analyses were conducted within the TriNetX platform environment. Missing data were not imputed. Laboratory variables were analyzed using a complete-case approach, where each test was assessed using only available observations. The “% of cohort” column in Table 1 reflects the proportion of patients with non-missing values for each laboratory measurement, enabling transparent evaluation of data completeness. Follow-up time was uniformly calculated from the index date, and right censoring was applied to account for loss to follow-up in survival analyses.

## 3. Results

**Figure 2:**
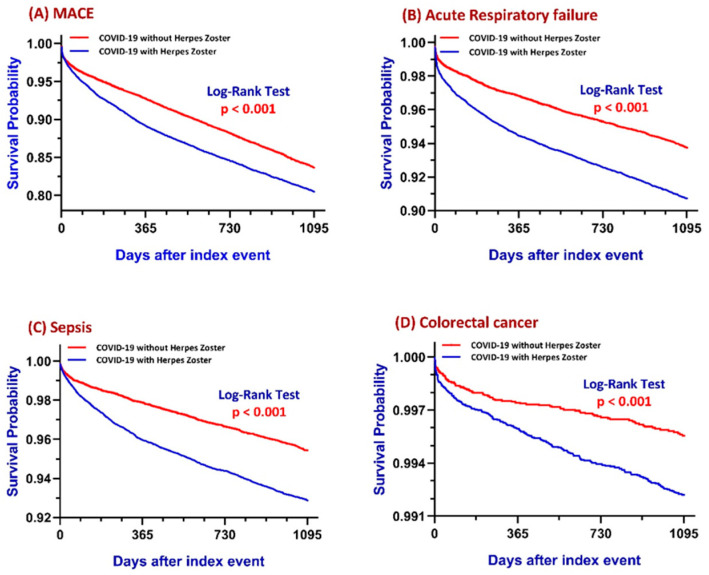
Comprehensive 3-year risk analysis of major adverse cardiovascular events (MACE) (**A**), acute respiratory failure (ARF) (**B**), sepsis (**C**), and colorectal cancer disease-free rate (**D**).

Figure 2 summarizes a comprehensive 3-year risk analysis of major adverse cardiovascular events (MACE), acute respiratory failure (ARF), sepsis, and colorectal cancer among COVID-19 patients with and without herpes zoster.

### 3.1. Major Adverse Cardiovascular Events (MACE)

The analytic cohorts consisted of 18,818 patients with HZ and 20,426 patients without HZ. Risk analysis shows a higher incidence of MACE in the COVID+HZ group (17.7%) compared to the COVID-HZ group (11.4%). This translates into a risk difference of 6.3% (95% CI: 5.6–7.0%, z = 17.734, *p* < 0.001), a risk ratio of 1.152, and an odds ratio of 1.671, indicating a 55% to 67% increased likelihood of developing MACE in patients with HZ. (Supplementary Table S2)

KM survival analysis further supports these findings (Figure 2A). The survival probability at the end of the 3-year period was 80.79% for the COVID+HZ group and 84.79% for the COVID-HZ group. The difference in time-to-event trajectories between the two groups was statistically significant, as demonstrated by the log-rank test (χ^2^ = 105.666, *p* < 0.001). The hazard ratio for MACE was 1.319 (95% CI: 1.251–1.391), with the proportionality assumption statistically validated (χ^2^ = 25.409, *p* < 0.001), indicating a 31.9% higher instantaneous risk of cardiovascular events over the study period in the HZ group.

**Figure 3:**
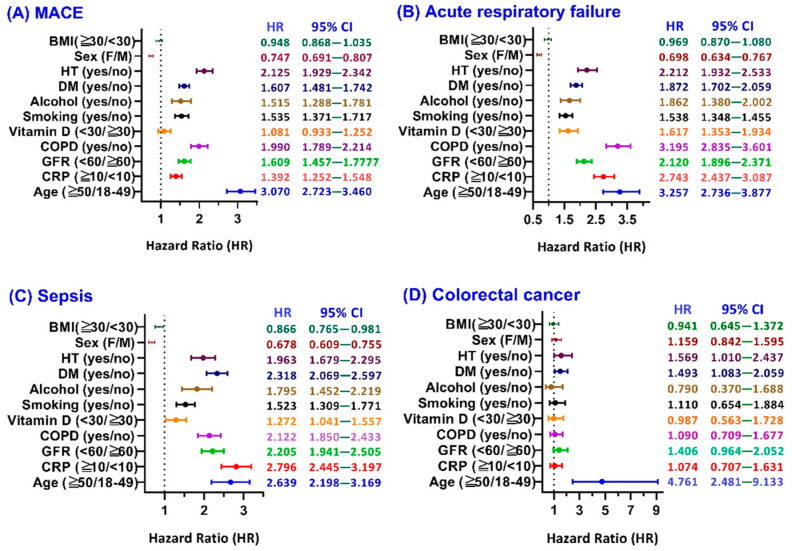
Subgroup analysis of 3-year risk for (**A**) major adverse cardiovascular events (MACE); (**B**) acute respiratory failure (ARF); (**C**) sepsis; and (**D**) colorectal cancer in COVID-19 patients with or without HZ. The vertical dashed line indicates a hazard ratio of 1.0, representing no difference in risk between groups.

Subgroup analysis (Figure 3A) in the forest plot among COVID-19 patients with HZ show patients aged ≥50 years had a markedly elevated HR of 3.07 (95% CI: 2.723–3.460), indicating a more than threefold increased risk of MACE compared to those aged 18–49, highlighting age as a major risk amplifier. Similarly, those with impaired kidney function (GFR < 60 mL/min) had an HR of 1.609 (95% CI: 1.457–1.777), while patients with COPD exhibited a nearly twofold increased risk (HR: 1.99, 95% CI: 1.789–2.214), reflecting the compounding effect of systemic comorbidities. Elevated CRP (≥10 mg/dL) was associated with a significantly increased HR of 1.392 (95% CI: 1.252–1.548), underscoring the contribution of inflammation to cardiovascular complications. Other notable risk-enhancing covariates included vitamin D deficiency (HR: 1.081), smoking (HR: 1.535), alcohol dependence (HR: 1.515), diabetes mellitus (HR: 1.607), and hypertension (HR: 2.125), all of which showed statistically significant associations. Conversely, the females were associated with a lower risk compared to the males (HR: 0.747, 95% CI: 0.691–0.807), and BMI ≥ 30 showed a neutral effect (HR: 0.948, 95% CI: 0.868–1.035).

### 3.2. Acute Respiratory Failure (ARF)

This analytic cohort consisted of 25,487 patients in the COVID+HZ group and 25,721 in the COVID-HZ group. COVID+HZ with 2200 developing respiratory failure (8.6%); however, in the COVID-HZ group, 1285 developed respiratory failure (5.0%). Risk Analysis yielded a risk difference of 3.6% (95% CI: 3.2–4.1%, z = 16.335, *p* < 0.001), confirming a statistically significant increase in absolute risk for respiratory failure in the HZ group. The risk ratio was 1.728 (95% CI: 1.616–1.847), and the odds ratio was 1.797 (95% CI: 1.673–1.929), indicating that COVID-19 patients who developed HZ were approximately 73–80% more likely to experience respiratory failure compared to those who did not. (Supplementary Table S2)

Kaplan–Meier survival analysis (Figure 2B) reveals the finding that the presence of herpes zoster is associated with a 73–80% higher likelihood of developing ARF within three years following COVID-19 and shows a clear divergence in survival curves. At the end of the 3-year follow-up, the probability of remaining free from ARF was 90.72% in the COVID+HZ group and 93.64% in the COVID-HZ group. The difference was statistically significant, as confirmed by the log-rank test (χ^2^ = 132.750, *p* < 0.001). The estimated hazard ratio was 1.495 (95% CI: 1.395–1.601), indicating a 49.5% higher instantaneous risk of ARF in the HZ group. The test for proportionality was marginal (χ^2^ = 3.648, *p* = 0.056), suggesting acceptable model fit.

Subgroup analyses (Figure 3B) in the forest plot show that patients aged ≥50 years had a significantly elevated risk of acute respiratory failure (HR: 3.257, 95% CI: 2.736–3.877), indicating a more than threefold increase compared to those aged 18–49. Elevated C-reactive protein (CRP ≥ 10 mg/dL) was also strongly associated with acute respiratory failure (HR: 2.743, 95% CI: 2.437–3.087), emphasizing the contribution of systemic inflammation. Similarly, impaired kidney function (GFR < 60 mL/min) conferred a high risk (HR: 2.120, 95% CI: 1.896–2.371). Among comorbid conditions, COPD showed a particularly strong association (HR: 3.195, 95% CI: 2.835–3.601), reflecting the synergistic respiratory burden of underlying pulmonary disease and herpes zoster. Additional elevated risks were observed in those with low vitamin D (HR: 1.617), smoking history (HR: 1.538), alcohol dependence (HR: 1.662), diabetes mellitus (HR: 1.872), and hypertension (HR: 2.212)—all with statistically significant confidence intervals. Notably, female sex appeared to confer a protective effect compared to males (HR: 0.698, 95% CI: 0.634–0.767), while BMI ≥ 30 had a neutral impact (HR: 0.969, 95% CI: 0.870–1.080).

### 3.3. Sepsis

The analytic cohorts included 25,966 patients in the COVID+HZ group and 26,389 in the COVID-HZ group. The crude incidence of sepsis was significantly higher in the COVID+HZ group (6.4%) compared to the COVID-HZ group (3.6%). Risk analysis reveals the risk difference of 2.8% (95% CI: 2.5–3.2%, z = 14.908, *p* < 0.001), a risk ratio of 1.795 (95% CI: 1.660–1.941), and an odds ratio of 1.849 (95% CI: 1.704–2.007), indicating that COVID-19 patients who later developed HZ were approximately 80% more likely to experience sepsis over the 3-year follow-up period. (Supplementary Table S2)

KM survival analysis (Figure 2C) reinforced these findings by illustrating the time-dependent nature of sepsis onset. At the end of the observation window, the survival probability free from sepsis at the end of the 3-year period was 93.07% for the COVID+HZ cohort and 95.34% for the COVID-HZ cohort. The log-rank test confirmed a statistically significant difference in survival curves (χ^2^ = 110.279, *p* < 0.001). The estimated hazard ratio was 1.530 (95% CI: 1.413–1.658), with the proportional hazards’ assumption supported (χ^2^ = 12.134, *p* < 0.001), indicating a 53% higher instantaneous risk of sepsis in patients who developed HZ. These findings demonstrate a strong and clinically meaningful association between HZ and elevated long-term sepsis risk in COVID-19 survivors, emphasizing the need for proactive monitoring and preventive strategies in this vulnerable patient population.

The subgroup analysis (Figure 3C) in the forest plot demonstrates that all examined covariates, except for sex and BMI, are associated with an increased risk of sepsis in the presence of HZ. Patients aged ≥50 years had a significantly elevated risk (HR: 2.639; 95% CI: 2.198–3.169), as did those with CRP ≥ 10 mg/dL (HR: 2.796; 95% CI: 2.445–3.197), reflecting the central role of age and inflammation. Impaired kidney function (GFR < 60 mL/min) was also a strong predictor (HR: 2.205; 95% CI: 1.941–2.505), which is consistent with known immunocompromised states in chronic kidney disease. Patients with COPD (HR: 2.122), vitamin D deficiency (HR: 1.272), smoking history (HR: 1.523), alcohol dependence (HR: 1.795), diabetes mellitus (HR: 2.318), and hypertension (HR: 1.963) all exhibited elevated risk with narrow confidence intervals, indicating statistical robustness. Interestingly, female sex was associated with a lower sepsis risk compared to males (HR: 0.678; 95% CI: 0.609–0.755), and BMI ≥ 30 showed a modestly reduced risk (HR: 0.866; 95% CI: 0.765–0.981), suggesting possible protective metabolic or immunological factors that warrant further investigation.

### 3.4. Colorectal Cancer (CRC)

This final analytic cohorts of 27,258 patients in the COVID+HZ group and 27,313 in the COVID-HZ group. The incidence of colon cancer was higher in in the COVID+HZ group (0.7%) compared to the COVID-HZ group (0.4%). This corresponded to a risk difference of 0.3% (95% CI: 0.2–0.4%, z = 4.604, *p* < 0.001), a risk ratio of 1.711 (95% CI: 1.358–2.157), and an odds ratio of 1.716 (95% CI: 1.360–2.166), indicating that individuals who developed herpes zoster after COVID-19 had approximately 71% higher odds of developing colorectal cancer over the 3-year follow-up period (Supplementary Table S2).

KM survival analysis (Figure 2D) provided additional insight into the timing of cancer onset. At the end of the follow-up period, the probability of remaining free from colon cancer was 99.22% in the COVID+HZ cohort and 99.46% in the COVID-HZ cohort. Despite the low absolute incidence, the difference in time-to-event distribution was statistically significant, as shown by the log-rank test (χ^2^ = 9.955, df = 1, *p* = 0.002). The hazard ratio (HR) was 1.450 (95% CI: 1.150–1.829), indicating a 45% higher instantaneous risk of colorectal cancer in the COVID+HZ group. The proportional hazards assumption was satisfied (χ^2^ = 0.254, *p* = 0.614), validating the model.

Subgroup analysis (Figure 3D) in the forest plot among the covariates assessed, age emerged as the most significant factor, with patients aged ≥50 showing a dramatically higher risk of colorectal cancer (HR: 4.761; 95% CI: 2.481–9.133) compared to those aged 18–49, highlighting age as a major driver of cancer risk. Chronic kidney impairment (GFR < 60 mL/min) was also associated with a higher risk (HR: 1.406; 95% CI: 0.964–2.052), suggesting a possible link between renal dysfunction and cancer susceptibility, although the confidence interval crosses 1.0, indicating borderline significance. Similarly, patients with diabetes mellitus (HR: 1.493; 95% CI: 1.083–2.059) and hypertension (HR: 1.569; 95% CI: 1.010–2.437) showed statistically significant elevations in risk. In contrast, variables such as elevated CRP (HR: 1.074), COPD (HR: 1.090), vitamin D deficiency (HR: 0.987), smoking (HR: 1.110), and sex (female vs. male, HR: 1.159) did not show statistically significant effects, as their 95% confidence intervals crossed 1.0. Interestingly, alcohol dependence (HR: 0.790; 95% CI: 0.370–1.688) and obesity (BMI ≥ 30, HR: 0.941; 95% CI: 0.645–1.372) were not associated with increased risk and may reflect unmeasured confounders or sample size limitations.

### 3.5. Sensitivity Analysis

To evaluate the robustness of our findings, we conducted multiple sensitivity analyses. First, we assessed outcome risks using Kaplan–Meier survival analysis before and after propensity score matching (PSM). Across all outcomes—major adverse cardiovascular events (MACE), acute respiratory failure (ARF), sepsis, and colorectal cancer (CRC)—COVID-19 patients who developed herpes zoster (HZ) showed significantly higher risks compared to those without HZ. These associations remained consistent and statistically significant following PSM, indicating that baseline differences were adequately controlled (see Supplementary Table S3).

Second, to rule out potential confounding from pre-existing immune dysfunction, we performed a sensitivity analysis that excluded patients with documented immunosuppressive conditions or treatments within one year prior to COVID-19. ICD-10 codes used to define immunosuppression included Z79.62, Z79.899, D84.821, D89.8, and D90. As shown in Supplementary Table S4, the associations between post-COVID HZ and adverse outcomes remained significant, further supporting the validity of our findings.

## 4. Discussion

Emerging studies suggest that COVID-19 may disrupt immune surveillance mechanisms essential for identifying and eliminating nascent cancer cells [15]. While initial research focused primarily on the respiratory effects of the virus, increasing attention has shifted toward its broader systemic impacts, including potential links to oncogenesis. SARS-CoV-2 contributes to immune dysregulation, chronic inflammation, and tissue damage, all of which may facilitate tumor development. Although no studies have directly established colorectal cancer (CRC) as uniquely sensitive to post-viral immune dysregulation, CRC is known to depend heavily on mucosal immune integrity and localized immune surveillance. Given the overlap between immune exhaustion observed in chronic viral infections and the immune escape features seen in CRC, our findings raise a biologically plausible hypothesis that warrants further investigation. In our analysis, COVID+HZ patients demonstrated a 45% increased risk of developing CRC. Subgroup analysis identified age ≥ 50 as the most significant risk factor, with chronic kidney impairment, diabetes, and hypertension also contributing to elevated risk.

The virus uses the ACE2 receptor for cell entry, which is found not only in the lungs but also in the gastrointestinal tract and other tissues involved in oncologic processes, such as the colorectal epithelium [16]. This raises concerns about tissue-specific vulnerability and long-term changes that may contribute to tumorigenesis. Observational studies have begun to examine potential links between post-COVID conditions and an increased incidence or severity of cancers, including colorectal and hematological malignancies. While a direct causal relationship has not been definitively established, studies have noted worse cancer outcomes and staging [17,18]. Furthermore, the COVID-19 pandemic has disrupted colorectal cancer screening programs, leading to a significant decline in early detection rates, which has temporarily reduced reported CRC incidence, potentially skewing the true prevalence of the disease. In addition to screening disruptions, biological mechanisms may also contribute to increased CRC risk in this population. Prior studies have demonstrated that chronic inflammation and impaired T-cell-mediated immune surveillance may promote colorectal tumorigenesis that may be exacerbated in the setting of post-COVID immune dysregulation and herpes zoster reactivation [19,20].

Studies have explored the link between COVID-19 and latent varicella-zoster virus reactivation, with a review identifying 27 HZ cases and a cohort study showing increased risk, highlighting the need for monitoring HZ in COVID-19 patients [21]. Immune suppression significantly contributes to colorectal cancer development, as it impairs the body’s ability to detect and eliminate abnormal cells, increasing cancer risk [22]. We aim to explore whether COVID-19 and herpes zoster (HZ) co-infection exacerbates immune suppression and colorectal cancer risk. TriNetX, a global network with data from 300 million patients, enables large-scale analyses of inflammatory diseases and cancer outcomes. Due to data extraction limitations from TriNetX, we restricted the cohort to patients aged 55–60 years and performed propensity score matching (PSM) to ensure feasibility. While PSM minimized demographic disparities, it reduced population diversity, particularly among minorities, and left residual imbalances in comorbidities, medication use, and inflammatory markers, affecting external validity and clinical generalizability.

Our study highlights a significant association between HZ superinfection and increased cardiovascular risk in COVID-19 survivors, emphasizing the need for cardiovascular monitoring. Older age, cardiometabolic comorbidities, inflammation, and lifestyle factors further exacerbate this risk, supporting targeted monitoring and early intervention in high-risk subgroups. Another key observation shows a significantly higher risk of acute respiratory failure (ARF) in COVID-19 patients with herpes zoster (HZ), with lower survival probabilities. Key predictors include COPD, age, elevated CRP levels, and impaired renal function, emphasizing the impact of inflammation, organ dysfunction, and aging on respiratory outcomes.

Given that published studies have shown that immunological dysfunction can persist long after SARS-CoV-2 infection [23]. Our analysis shows that COVID-19 patients with herpes zoster (HZ) superinfection have a significantly higher incidence of sepsis, suggesting a worse immune status. Elevated CRP levels emerged as a key risk factor, highlighting the need for heightened monitoring and preventive care in high-risk post-COVID individuals. Recent literature has also delineated potential biological mechanisms through which SARS-CoV-2 may influence oncogenesis. These include persistent low-grade inflammation, cytokine dysregulation, and activation of oncogenic signaling pathways such as NF-κB, JAK–STAT, and MAPK, all of which are implicated in colorectal cancer development [2,5,15]. In addition, viral proteins such as spike and NSPs may disrupt tumor suppressor pathways and DNA repair machinery [5]. The prolonged immune exhaustion affecting T-cell and NK-cell function [23] further supports a mechanistic link between post-infectious immune dysregulation and impaired tumor surveillance, particularly in patients with HZ. These observations provide a biologically plausible framework that complements the epidemiologic associations observed in this study.

Due to disruptions in routine colorectal cancer (CRC) screenings and colonoscopies during the COVID-19 pandemic, there has been a decline in new CRC diagnoses, leading to potential underreporting of true incidence. Our analysis focused solely on COVID-19 patients, using Kaplan–Meier survival analysis to evaluate CRC disease-free rates. The results show a significant association between herpes zoster (HZ) and increased CRC risk in COVID-19 patients, with those co-infected or superinfected with COVID-19 and HZ having a higher risk over time, as indicated by the widening gap between the two survival curves. Given the immune-related mechanisms underlying CRC and its sensitivity to screening practices, pandemic-related reductions in screening intensity may have compounded biologically driven risks in vulnerable populations. Key risk factors for CRC, including hypertension, diabetes, and age ≥ 50, were identified, emphasizing the need for targeted surveillance and early diagnostic evaluation in high-risk groups.

This study has several limitations that should be considered when interpreting the findings. These may reflect unmeasured differences in inflammatory or nutritional status, potentially introducing bias. Second, the retrospective observational design limits causal inference; the associations between HZ and subsequent outcomes may be influenced by unmeasured confounders, and temporality alone does not establish causation. Although the following commenced immediately after the COVID-19 diagnosis, we recognize that immune effects on cancer development are unlikely to manifest during the acute phase. To address this, we performed a time-stratified sensitivity analysis separating early (1–90 days) and delayed (91 days to 3 years) follow-up intervals. The elevated CRC risk was primarily observed during the long-term period, although it was not statistically significant due to low absolute event counts. These findings are consistent with the delayed divergence of Kaplan–Meier curves (Figure 2D), supporting the hypothesis of a post-infectious, immune-related mechanism in colorectal tumorigenesis. Third, several important clinical factors were not available on the TriNetX platform and could not be adjusted for, including antiviral treatment for HZ, medication adherence, vaccination status (HZ or COVID-19), and COVID-19 severity (e.g., hospitalization or ICU admission). This absence limits comprehensive risk adjustment. Fourth, we encountered limitations related to data processing capabilities. Specifically, the platform’s constraints necessitated splitting patient cohorts and implementing age-restricted matching; this approach may affect the generalizability of our findings to broader age groups.

## 5. Conclusions

In this real-world multicenter cohort study, we found that herpes zoster (HZ) following COVID-19 infection may indicate broader immune dysfunction and long-term health risks. Viral reactivation may reflect deeper immune compromise, impairing the body’s ability to control inflammation, infection, and tumor development. Patients with post-COVID HZ had significantly higher risks of cardiovascular events, sepsis, and colorectal cancer, with this trend persisting over 3 years, especially in older adults and those with comorbidities. Our findings suggest that HZ could serve as a new risk marker for earlier screening, highlighting the need for clinicians to consider post-COVID HZ as a signal of immune vulnerability and prioritize aggressive colonoscopy examination.

## Figures and Tables

**Table 1:** Selected baseline characteristics after Propensity Score Matching (Age 55–60). All baseline variables before and after matching are provided.

	Before Matching				After Matching			
Characteristics	COVID-19 with HZ (*n* = 3155)		COVID-19 Without HZ (*n* = 963,799)		COVID-19 with HZ (*n* = 3154)		COVID-19 Without HZ (*n* = 3154)	
	+HZ	−HZ	*p*-Value	Std Diff.	+HZ	−HZ	*p*-Value	Std Diff.
	% of Cohort	% of Cohort			% of Cohort	% of Cohort		
Demographics (Mean ± SD)								
Age	with HZ		without HZ		with HZ		without HZ	
	53.1 ± 1.8		53.1 ± 1.8		53.1 ± 1.8		53.1 ± 1.9	
	+HZ	−HZ	0.460	0.013	+HZ	−HZ	0.919	0.003
	100%	100%			100%	100%		
White	71.0%	57.9%	<0.001	0.277	71.0%	72.0%	0.403	0.021
Female	66.5%	54.4%	<0.001	0.250	66.5%	66.6%	0.894	0.003
Black or African American	11.7%	15.0%	<0.001	0.096	11.7%	11.3%	0.581	0.014
Male	33.5%	45.6%	<0.001	0.250	33.5%	33.4%	0.894	0.003
Asian	4.8%	4.1%	0.051	0.033	4.8%	4.7%	0.906	0.003
**Diagnosis**								
	+HZ	−HZ	*p*-Value	Std diff.	+HZ	−HZ	*p*-Value	Std diff.
Diabetes mellitus	14.3%	7.5%	<0.001	0.218	14.2%	14.0%	0.800	0.006
Hypertensive diseases	25.4%	15.0%	<0.001	0.261	25.4%	25.7%	0.795	0.007
Cerebrovascular diseases	2.0%	1.2%	<0.001	0.061	2.0%	1.9%	0.855	0.005
Noninfective enteritis and colitis	2.7%	1.1%	<0.001	0.117	2.7%	2.5%	0.692	0.010
Ischemic heart diseases	4.0%	2.8%	<0.001	0.067	4.0%	3.5%	0.288	0.027
**Medication**								
	+HZ	−HZ	*p*-Value	Std diff.	+HZ	−HZ	*p*-Value	Std diff.
BETA BLOCKERS/RELATED	9.3%	5.7%	<0.001	0.136	9.3%	9.2%	0.862	0.004
DIURETICS	11.0%	6.3%	<0.001	0.166	11.0%	10.3%	0.414	0.021
CALCIUM CHANNEL BLOCKERS	7.1%	4.5%	<0.001	0.114	7.1%	6.8%	0.585	0.014
ACE INHIBITORS	6.7%	4.6%	<0.001	0.093	6.7%	6.8%	0.802	0.006
ANGIOTENSIN II INHIBITOR	5.8%	3.6%	<0.001	0.106	5.8%	5.9%	0.914	0.003
ALPHA BLOCKERS	2.1%	1.4%	<0.001	0.059	2.1%	1.9%	0.472	0.018
BLOOD GLUCOSE REGULATION AGENTS	12.8%	6.8%	<0.001	0.203	12.8%	11.8%	0.206	0.032
ANTILIPEMIC AGENTS	13.2%	8.3%	<0.001	0.159	13.2%	13.1%	0.941	0.002
**Laboratory** (Mean ± SD)								
	with HZ		without HZ		with HZ		without HZ	
	+HZ, % of Cohort	−HZ, % of Cohort	*p*-Value	Std diff.	+HZ, % of Cohort	−HZ, % of Cohort	*p*-Value	Std diff.
Ferritin, ng/mL	368.3 ± 767.2		290.1 ± 828.5		368.3 ± 767.2		240.8 ± 404.6	
	4.5%	2.6%	0.262	0.098	4.5%	4.9%	0.072	0.208
C reactive protein, mg/L	16.6 ± 47.1		23.9 ± 50.3		16.7 ± 47.3		19.7 ± 43.2	
	6.0%	3.2%	0.045	0.151	6.0%	6.5%	0.517	0.065
Sodium, mmol/L	138.8 ± 3.0		139.0 ± 3.0		138.8 ± 3.0		139.0 ± 3.0	
	43.1%	26.1%	0.029	0.059	43.1%	43.1%	0.191	0.050
Potassium, mmol/L	4.1 ± 0.5		4.2 ± 0.5		4.1 ± 0.5		4.2 ± 0.4	
	42.3%	25.5%	0.230	0.033	42.3%	42.3%	0.203	0.049
Urea nitrogen, mg/dL	17.1 ± 11.8		16.2 ± 9.5		17.1 ± 11.8		16.2 ± 9.2	
	40.8%	23.0%	0.001	0.083	40.7%	40.4%	0.032	0.085
Creatinine, mg/dL	1.1 ± 1.3		1.0 ± 1.4		1.1 ± 1.3		1.0 ± 1.0	
	43.1%	25.7%	0.450	0.022	43.1%	42.9%	0.148	0.056
Glucose, mg/dL	119.6 ± 58.8		117.4 ± 55.3		119.6 ± 58.8		120.6 ± 58.2	
	42.1%	25.8%	0.154	0.038	42.1%	2.4%	0.674	0.016
Calcium, mg/dL	9.4 ± 0.6		9.3 ± 0.6		9.4 ± 0.6		9.4 ± 0.5	
	41.9%	24.3%	0.145	0.040	41.9%	41.6%	0.730	0.013
Hemoglobin, g/dL	13.3 ± 2.0		13.5 ± 2.0		13.3 ± 2.0		13.4 ± 1.9	
	39.8%	23.5%	<0.001	0.120	39.8%	39.8%	0.172	0.055
ALT, U/L	28.0 ± 23.2		31.0 ± 69.4		28.0 ± 23.2		28.3 ± 21.3	
	37.5%	21.7%	0.129	0.059	37.5%	37.8%	0.755	0.013
AST, U/L	27.3 ± 25.0		30.1 ± 94.6		27.3 ± 25.0		27.1 ± 22.0	
	37.4%	21.3%	0.307	0.041	37.4%	37.5%	0.829	0.009
Bilirubin.total, mg/dL	0.6 ± 1.6		0.6 ± 1.0		0.6 ± 1.6		0.6 ± 1.1	
	35.7%	20.3%	0.433	0.018	35.7%	35.9%	0.768	0.012
Albumin, g/dL	4.1 ± 0.5		4.1 ± 0.5		4.1 ± 0.5		4.1 ± 0.5	
	36.3%	20.7%	0.913	0.003	36.3%	36.3%	0.363	0.038
Cholesterol, mg/dL	192.2 ± 45.0		190.7 ± 45.1		192.2 ± 45.1		195.0 ± 43.6	
	22.2%	12.2%	0.371	0.034	22.2%	22.3%	0.239	0.063
Cholesterol in LDL, mg/dL	110.6 ± 37.2		110.8 ± 37.8		110.6 ± 37.2		113.6 ± 38.1	
	21.7%	12.3%	0.924	0.004	21.7%	21.7%	0.149	0.078
Cholesterol in HDL, mg/dL	50.9 ± 21.0		49.5 ± 19.8		50.9 ± 21.0		49.5 ± 20.6	
	22.0%	12.5%	0.063	0.069	22.0%	22.1%	0.211	0.067
Triglyceride, mg/dL	146.2 ± 107.5		149.1 ± 136.1		146.2 ± 107.5		154.9 ± 110.0	
	21.7%	12.5%	0.580	0.024	21.7%	22.3%	0.138	0.080
Hemoglobin A1c/Hemoglobin.total	6.7 ± 1.9		6.6 ± 1.8		6.7 ± 1.9		6.7 ± 1.8	
	18.3%	10.1%	0.107	0.066	18.3%	17.5%	0.875	0.009
Calcidiol, ng/mL	35.4 ± 18.0		33.4 ± 17.1		35.4 ± 18.0		34.0 ± 17.7	
	5.0%	2.3%	0.139	0.115	5.0%	5.0%	0.491	0.078
Iron, ug/dL	73.0 ± 47.4		74.5 ± 44.2		73.0 ± 47.4		77.4 ± 53.7	
	4.3%	2.3%	0.695	0.032	4.3%	4.6%	0.465	0.087

## Data Availability

The data presented in this study are available on request from the corresponding author due to privacy and ethical restrictions. The dataset was obtained from the TriNetX global federated health research network, which collects deidentified electronic medical records from multiple healthcare institutions. Access to the dataset is restricted by institutional policies and data-sharing agreements. Researchers interested in accessing the data may request it from TriNetX, subject to institutional approval and compliance with data privacy regulations.

## Supplementary Materials

The following supporting information can be downloaded at: https://www.mdpi.com/article/10.3390/cancers17142306/s1, Supplementary Table S1, Comparison of 3-year outcome risk between the age-restricted (55–60 years) and full adult (18–89 years) COVID-19+HZ cohorts, showing similar hazard ratios across outcomes including ARF, CRC, and MACE. Supplementary Table S2, Comparative 3-year risk assessment of MACE, ARF, sepsis, and colorectal cancer in COVID-19 patients with versus without herpes zoster (HZ). Supplementary Table S3, Hazard ratios for major outcomes before and after propensity score matching (PSM), derived from Kaplan–Meier survival analyses. Supplementary Table S4, Sensitivity analysis excluding patients with documented immunosuppression prior to COVID-19, confirming robustness of associations. Supplementary Table S5, Time-stratified analysis of colorectal cancer risk (1–90 days vs. 91 days to 3 years) following COVID-19 and HZ, supporting long-term immune-related contributions.
