# Supplementary material for: Colorectal Cancer Risk Following Herpes Zoster Reactivation in COVID-19 Survivors: Global Multicenter Study Using TriNetX
Source: Cancers (Basel). 2025 Jul 11;17(14):2306. doi: 10.3390/cancers17142306 (PMC12293121; doi:10.3390/cancers17142306)
Supplement: Supplementary file 1 [file cancers-17-02306-s001.zip › cancers-3699539-supplementary.pdf]

Supplementary Materials:

Supplementary Table S1

Outcomes	HR	95% CI, low	95% CI, high	P value Log-Rank Test
MACE	1.004	0.811	1.244	0.970
ARF	1.014	0.735	1.398	0.934
Sepsis	0.994	0.701	1.409	0.971
CRC	1.124	0.434	2.912	0.810

To evaluate the 3-year clinical outcome risk, we compared hazard ratios (HRs) derived from Kaplan-Meier survival analyses between two cohorts: (1) an age-restricted group (55-60 years) with COVID-19 and herpes zoster (HZ), and (2) the full cohort (18–89 years) of COVID-19 patients with HZ. ARF: Acute Respiratory Failure, CRC: Colorectal cancer, MACE: Major Adverse Cardiovascular Event. Across all outcomes, the hazard ratios in the 55-60 age subgroup were close to 1 and not statistically significant, suggesting no increased or decreased risk relative to the broader adult population. These findings indicate that in patients aged 55-60, the risk patterns for adverse outcomes following HZ in the setting of COVID-19 are comparable to those observed in the full adult cohort.

Supplementary Table S2

Outcomes	Cohorts	Risk	Risk Difference	Risk Ratio	Odds Ratio	Z value	P value
MACE	COVID + HZ	0.177	0.063	1.152	1.671	17.734	< 0.0001
	COVID - HZ	0.114					
ARF (Acute Respiratory Failure)	COVID + HZ	0.086	0.036	1.728	1.797	16.335	< 0.0001
	COVID - HZ	0.050					
Sepsis	COVID + HZ	0.064	0.028	1.795	1.849	14.908	< 0.0001
	COVID - HZ	0.036					
Colorectal cancers	COVID + HZ	0.007	0.003	1.711	1.716	4.604	< 0.0001
	COVID - HZ	0.004					

The table presents a comparative 3-year risk assessment of four major clinical outcomes—MACE (major adverse cardiovascular events), ARF (acute respiratory failure), sepsis, and colorectal cancers—in COVID-19 patients with versus without herpes zoster (HZ).

Supplementary Table S3

Outcomes	PSM	HR	95% CI, low	95% CI, high	P value Log-Rank Test
MACE	Before	1.900	1.183	1.966	< 0.0001
	After	1.295	1.228	1.365	< 0.0001
ARF	Before	2.212	2.121	2.306	< 0.0001
	After	1.534	1.431	1.645	< 0.0001
Sepsis	Before	2.389	2.277	2.507	< 0.0001
	After	1.647	1.517	1.788	< 0.0001
Colorectal cancer	Before	2.142	1.862	2.466	< 0.0001
	After	1.376	1.095	1.729	< 0.01

3-year clinical outcome risk by analyzing hazard ratios derived from Kaplan–Meier survival curves before and after propensity score matching. ARF: Acute Respiratory Failure, MACE: Major Adverse Cardiovascular Event. Hazard ratios (HRs) were derived from Kaplan–Meier survival curves both before and after propensity score matching (PSM), offering insight into the consistency and robustness of the associations while controlling for baseline differences.

Supplementary Table S4

Exclude immuno-suppression*	Outcomes	Cohorts	Patients in cohort	Patients with outcome	Survival probability at end of time window	Hazard Ratio	95% CI	Log-Rank test P value
Yes	MACE	COVID-19 + HZ	17,098	2,777	82.37%	1.337	(1.260, 1.417)	0.000
		COVID-19 - HZ	18,793	1,865	86.17%			
No		COVID-19 + HZ	18,818	3,328	80.79%	1.295	(1.228, 1.365)	0.000
		COVID-19 - HZ	20,355	2,359	84.49%			
Yes	Acute Respiratory Failure	COVID-19 + HZ	22,080	1,701	91.73%	1.537	(1.419, 1.665)	0.002
		COVID-19 - HZ	22,362	935	94.46%			
No		COVID-19 + HZ	25,487	2,200	90.72%	1.534	(1.431, 1.645)	0.000
		COVID-19 - HZ	25,755	1,253	93.75%			
Yes	Sepsis	COVID-19 + HZ	22,444	1,261	93.91%	1.671	(1.518, 1.839)	0.007
		COVID-19 - HZ	22,745	627	96.25%			
No		COVID-19 + HZ	25,966	1,660	93.07%	1.647	(1.517, 1.788)	0.000
		COVID-19 - HZ	26,379	872	95.62%			
Yes	Colorectal Cancer	COVID-19 + HZ	23,208	155	99.27%	1.329	(1.029, 1.716)	0.500
		COVID-19 - HZ	23,334	95	99.44%			
No		COVID-19 + HZ	27,258	193	99.22%	1.376	(1.095, 1.729)	0.006
		COVID-19 - HZ	27,347	119	99.43%			

Sensitivity analysis by excluding patients with immunosuppression within one year prior to COVID-19. To assess the robustness of the findings, we conducted a sensitivity analysis excluding individuals with documented immunosuppressive conditions or therapies within one year prior to COVID-19 diagnosis, based on ICD-10 codes including Z79.62, Z79.899, D84.821, D89.8, and D90. After removing these patients, the associations between post-COVID herpes zoster and major adverse outcomes, including MACE, ARF, sepsis, and colorectal cancer, remained statistically significant, suggesting that the results are not confounded by pre-existing immune dysfunction.

Supplementary Table S5

Time window	Outcome	Cohorts	Patients in cohort	Patients with outcome	Survival probability at end of time window	Hazard Ratio	95% CI	Log-Rank test P value
1-90 days	MACE	COVID-19 + HZ vs COVID-19 - HZ	18,428 vs 19,948	897 vs 630	95.10% vs 96.45%	1.375	(1.242, 1.523)	<0.0001
91 days-3 years	MACE	COVID-19 + HZ vs COVID-19 - HZ	17,458 vs 19,194	2,306 vs 1,640	85.29% vs 87.86%	1.256	(1.179, 1.338)	<0.0001
1-90 days	Acute respiratory failure	COVID-19 + HZ vs COVID-19 - HZ	24,685 vs 25,014	734 vs 415	97.01% vs 98.16%	1.629	(1.444, 1.837)	<0.0001
91 days-3 years	Acute respiratory failure	COVID-19 + HZ vs COVID-19 - HZ	23,861 vs 24,532	1,367 vs 832	93.64% vs 95.32%	1.397	(1.281, 1.523)	<0.0001
1-90 days	Sepsis	COVID-19 + HZ vs COVID-19 - HZ	25,226 vs 25,630	431 vs 217	98.28% vs 99.05%	1.813	(1.540, 2.135)	<0.0001
91 days-3 years	Sepsis	COVID-19 + HZ vs COVID-19 - HZ	24,705 vs 25,205	1,123 vs 645	94.95% vs 96.46%	1.467	(1.331, 1.616)	<0.0001
1-90 days	Colorectal cancer	COVID-19 + HZ vs COVID-19 - HZ	26,457 vs 26,542	56 vs 31	99.79% vs 99.87%	1.630	(1.051, 2.528)	<0.05
91 days-3 years	Colorectal cancer	COVID-19 + HZ vs COVID-19 - HZ	26,310 vs 26,416	114 vs 72	99.51% vs 99.62%	1.300	(0.968, 1.747)	0.080

Supplementary Table 5. Time-stratified analysis of colorectal cancer (CRC) risk in COVID-19 patients with or without herpes zoster (HZ). To explore temporal patterns of CRC development, we stratified the follow-up period into short-term (1–90 days) and long-term (91 days to 3 years) after COVID-19 diagnosis. The increased CRC risk among post-COVID HZ patients was mainly observed during the long-term period, though not statistically significant due to low event rates. These findings align with the delayed divergence seen in Kaplan–Meier curves (Figure 2D), supporting a potential long-term immunologic contribution to CRC development.
